# Accelerating Cancer Histopathology Workflows with Chemical Imaging and Machine Learning

**DOI:** 10.1158/2767-9764.CRC-23-0226

**Published:** 2023-09-18

**Authors:** Kianoush Falahkheirkhah, Sudipta S. Mukherjee, Sounak Gupta, Loren Herrera-Hernandez, Michael R. McCarthy, Rafael E. Jimenez, John C. Cheville, Rohit Bhargava

**Affiliations:** 1 Beckman Institute for Advanced Science and Technology, University of Illinois Urbana-Champaign, Urbana, Illinois.; 2 Department of Bioengineering, University of Illinois Urbana-Champaign, Urbana, Illinois.; 3 Laboratory Medicine and Pathology, Mayo Clinic, Rochester, Minnesota.; 4 Department of Chemical and Biomolecular Engineering, University of Illinois Urbana-Champaign, Urbana, Illinois.; 5 Department of Electrical and Computer Engineering, University of Illinois Urbana-Champaign, Urbana, Illinois.; 6 Mechanical Science and Engineering, University of Illinois Urbana-Champaign, Urbana, Illinois.; 7 Cancer Center at Illinois, University of Illinois Urbana-Champaign, Urbana, Illinois.

## Abstract

Histopathology has remained a cornerstone for biomedical tissue assessment for over a century, with a resource-intensive workflow involving biopsy or excision, gross examination, sampling, tissue processing to snap frozen or formalin-fixed paraffin-embedded blocks, sectioning, staining, optical imaging, and microscopic assessment. Emerging chemical imaging approaches, including stimulated Raman scattering (SRS) microscopy, can directly measure inherent molecular composition in tissue (thereby dispensing with the need for tissue processing, sectioning, and using dyes) and can use artificial intelligence (AI) algorithms to provide high-quality images. Here we show the integration of SRS microscopy in a pathology workflow to rapidly record chemical information from minimally processed fresh-frozen prostate tissue. Instead of using thin sections, we record data from intact thick tissues and use optical sectioning to generate images from multiple planes. We use a deep learning–based processing pipeline to generate virtual hematoxylin and eosin images. Next, we extend the computational method to generate archival-quality images in minutes, which are equivalent to those obtained from hours/days-long formalin-fixed, paraffin-embedded processing. We assessed the quality of images from the perspective of enabling pathologists to make decisions, demonstrating that the virtual stained image quality was diagnostically useful and the interpathologist agreement on prostate cancer grade was not impacted. Finally, because this method does not wash away lipids and small molecules, we assessed the utility of lipid chemical composition in determining grade. Together, the combination of chemical imaging and AI provides novel capabilities for rapid assessments in pathology by reducing the complexity and burden of current workflows.

**Significance:**

Archival-quality (formalin-fixed paraffin-embedded), thin-section diagnostic images are obtained from thick-cut, fresh-frozen prostate tissues without dyes or stains to expedite cancer histopathology by combining SRS microscopy and machine learning.

## Introduction

The identification and assessment of prostatic carcinoma is based on characteristic histomorphologic features observed by optical microscopy within prepared thin sections (∼5 µm, a single cell layer thick), cut onto glass slides prepared from processed tissue. In the path from tissue acquisition to thin sections, there are two principal methods namely, formalin-fixed paraffin embedded (FFPE) and fresh frozen (FF). Each of these have advantages and drawbacks that affect the ability to interpret structural changes and make accurate determinations. FFPE sections have relatively higher contrast and well-preserved morphology, making them the gold standard for clinical diagnoses. The extended processing required for FFPE sections, however, typically requires a wait of hours to days for diagnoses. Furthermore, solvents used for processing result in loss of lipids and small molecules. Formalin fixation induces molecular cross-links (1, 2) that make tissues highly stable and the results reproducible over long periods of time. However, this processing and cross-linking also renders samples suboptimal for molecular analyses particularly if the tissue is fixed in formalin for an extended period or as blocks age (3). FF processing, in contrast, preserves molecular content and needs minimal processing (in 15–30 minutes) that can make it the preferred method for intraoperative pathologic assessment. Preparing samples, however, requires significantly higher resources to be available and highly skilled practitioners. Importantly, slides produced from FF processing lack the quality of FFPE sections and have much higher incidence of sample preparation artifacts. These increase complexity in histomorphologic interpretation and lead to reduced diagnostic utility (4–7). Regardless of the processing method, tissue still needs to be stained for generating appreciable microscopic contrast between the different cell types and extracellular matrix. The most common stain used clinically is hematoxylin and eosin (H&E), in which protein-rich regions are stained pink (eosin) and nucleic acid–rich regions are stained blue (hematoxylin). In addition to requiring reagents and labor, intralaboratory and interlaboratory variations in staining (overstaining and understaining), and batch variations often affect assessment. These variations are especially problematic in applying digital methods and require development of normalization or correction algorithms (8, 9). Furthermore, H&E staining generally renders the stained tissue unavailable for other imaging/analytic modalities, a particular issue when small disease foci need to be examined in limited tissue materials. In this study, we address these issues by considering the specific case of prostate cancer. Prostate cancer is one of the most common male cancers in the United States, with estimates of more than a quarter million diagnoses annually and second only to lung cancer in terms of mortality (10). Prostate cancer is histologically assessed using the Gleason grading system (11), which is not only the strongest predictor of outcome in men with prostate cancer (12) but also one of the most durable and prototypical workflows in pathology. H&E images allow a distinction between epithelial cells and stroma as well as an appreciation of architectural glandular alterations used for cancer grading. Here we present a workflow for prostate cancer pathology that seeks to maintain the diagnostic quality of current workflows but considerably shortens the time and resources required for processing. Our approach is based on a combination of two emerging technologies—stimulated Raman scattering microscopy (SRSM) and deep learning (DL)—whose combined application seeks to rapidly generate high-quality, clinically-actionable images.

SRSM is a multiphoton optical microscopy technique that directly measures chemical composition of tissues arising from their Raman vibrational response while localizing the signal acquired from a region in tissue to dimensions of a few hundred nanometers. Since the pioneering work of the Xie group (13), SRSM has been applied to assess a variety of tissues like the brain (14–16), gastrointestinal sites (17, 18), larynx (19), pancreas (20), and prostate (21). In addition to label-free molecular measurement, SRSM also allows optical depth sectioning in thicker samples due to multiphoton localization. The use of near-infrared lasers allows SRSM to probe deeper in tissue compared with optical microscopy, avoid photobleaching that may affect fluorescence-based methods, and is applicable to thick, freshly excised tissue that limits mid-infrared (22–25) or ultraviolet microscopy (26). While near-infrared and optical approaches can also enable high-content three-dimensional pathology (27–29), our goal here was not to propose workflows that measure more data or provide new discovery tools but to decrease the time and effort to classical diagnostic histology images for evaluation by pathologists. With these considerations, we designed a workflow to record SRSM data using optical sectioning to assess large spatial regions of approximately single-cell-thick planes (i.e., virtual sectioning) from FF tissue samples that are considerably thicker than conventional microtome-cut sections. The resulting hyperspectral datasets cannot be directly interpreted by pathologists and, thus, require the use of artificial intelligence (AI) techniques (30). Relating to clinical and research practices, an active area for chemical imaging techniques is to use spectral information to generate realistic clinical images (31). Such spectra-to-stained image translation has been variously termed virtual, computational or stainless staining.

DL methods (32) are especially attractive for this task due to their powerful prediction capability and relative ease of deployment. Here we focus on a subclass of DL, namely generative adversarial networks (GAN; ref. 33), that have shown success in handling the nonlinear transformation between two different domains to synthesize realistic images (34). GANs have been widely applied for many digital histopathology tasks such as synthesizing histologic images (35–37), stain normalization (38, 39), and virtual staining (40–42). We build on these developments for the second part of our workflow where we focus on generating clinical (FF and FFPE) images from unstained tissues’ SRSM data. We first seek to generate H&E images from FF SRSM data. Second, from the virtual FF H&E images, we seek to generate virtual FFPE images. Virtual FF staining can enable faster cancer detection and assessment of tumor margins compared with current intraoperative protocols whereas generating gold standard diagnostic quality FFPE virtual images from the same data can provide images for reliable diagnoses much earlier than current practice, thereby uniting the benefits of both approaches in one workflow. The third part of our study is to assess the suitability of implementing our approach for routine prostate cancer histopathology. To assess the use of virtual images in practice, we quantified the quality of staining and quality of diagnoses by expert urologic pathologists.

## Materials and Methods

### Sample Preparation and Imaging

A total of 75 fresh FF prostate cancer tissue samples belonging to deidentified patients of clinically-relevant Gleason grades (3+3, 3+4, 4+3, 4+5, 5+4, and 5+5) were obtained from Mayo Clinic. Each sample was sequentially sectioned into 5 µm (stained with H&E for generating (stained with H&E for generating the real stain and for pathologist evaluation), 10 ìm and 50 ìm for generating the single-section and multi-section SRS data, respectively. This project was approved by Mayo Clinic and University of Illinois-Urbana Champaign Institutional Review Boards in accordance with the U.S. Common Rule. All samples were kept FF at −80°C. Before the experiments, the tissue samples were brought to room temperature under vacuum, rapidly fixed (<10 minutes) in 4% methanol-free paraformaldehyde solution (Electron Microscopy Sciences), washed in PBS buffer, water, and immediately imaged. This fixation step is not a necessity and was done to retain the tissue for subsequent staining with H&E for training and evaluating the model performance. The tissue processing involved no fat solvents and long term stability of the tissue was verified with H&E staining and evaluation after SRS imaging (>7 days).

All SRS images were measured with a custom upright SRS microscope (modified, Zeiss Axio Imager) described elsewhere (43). Briefly a dual output (1,064 nm/532 nm, Plecter Duo, APE) ultrafast (6 ps, 80 MHz repetition rate) oscillator was used to generate the two beams for SRS measurement. The 1,064 nm output (Stokes) was amplitude modulated at 5 MHz with an electro-optic modulator (Conoptics) and the 532 nm output was used to pump an optical parametric oscillator (Levante, APE), to generate a tunable pump frequency (800–820 nm) so that the difference frequency matches the vibrational modes of interest (2,800–3,200 cm^−1^).The two beams were spatially and temporally aligned on the sample, focused with a long working distance, near-infrared corrected achromatic water immersion objective (Zeiss, NA = 1.05, Magnification = 63X) raster scanned with a pair of galvanometer mirrors (Thorlabs) and the signal was detected with a large area custom built photodiode and demodulated at 5 MHz with a lock-in amplifier (Zurich Instruments). The Raman shift frequencies chosen in this article are 2,847, 2,879, 2,902, 2,933, 2,960, 2,979, and 3,062 cm^−1^ and hyperspectral images were measured from 2,800–3,105 cm^−1^ at 4 cm^−1^ frequency resolution. SRS images were measured at either 1 or 0.5 µm axial resolution and to perform the image registration images acquired at 1 µm are upsampled by factor of 2 using bilinear approach. A continuous focus tracking scheme based on a 4-point fitted tissue plane was used to overcome stage leveling inaccuracies. All SRS imaging was performed with fully hydrated tissue under water immersion. For a pixel dwell time of typically 100 µs, 1 mm × 1 mm tissue section sampled at 1 µm pixel size takes roughly 100 seconds per band. Hence, the total time to generate both virtual FF and FFPE images for a 1 mm^2^ region of interest (ROI) takes around 700 seconds+ 0.236 seconds (overall about 12 minutes).

### Model Design

**FIGURE 1:**
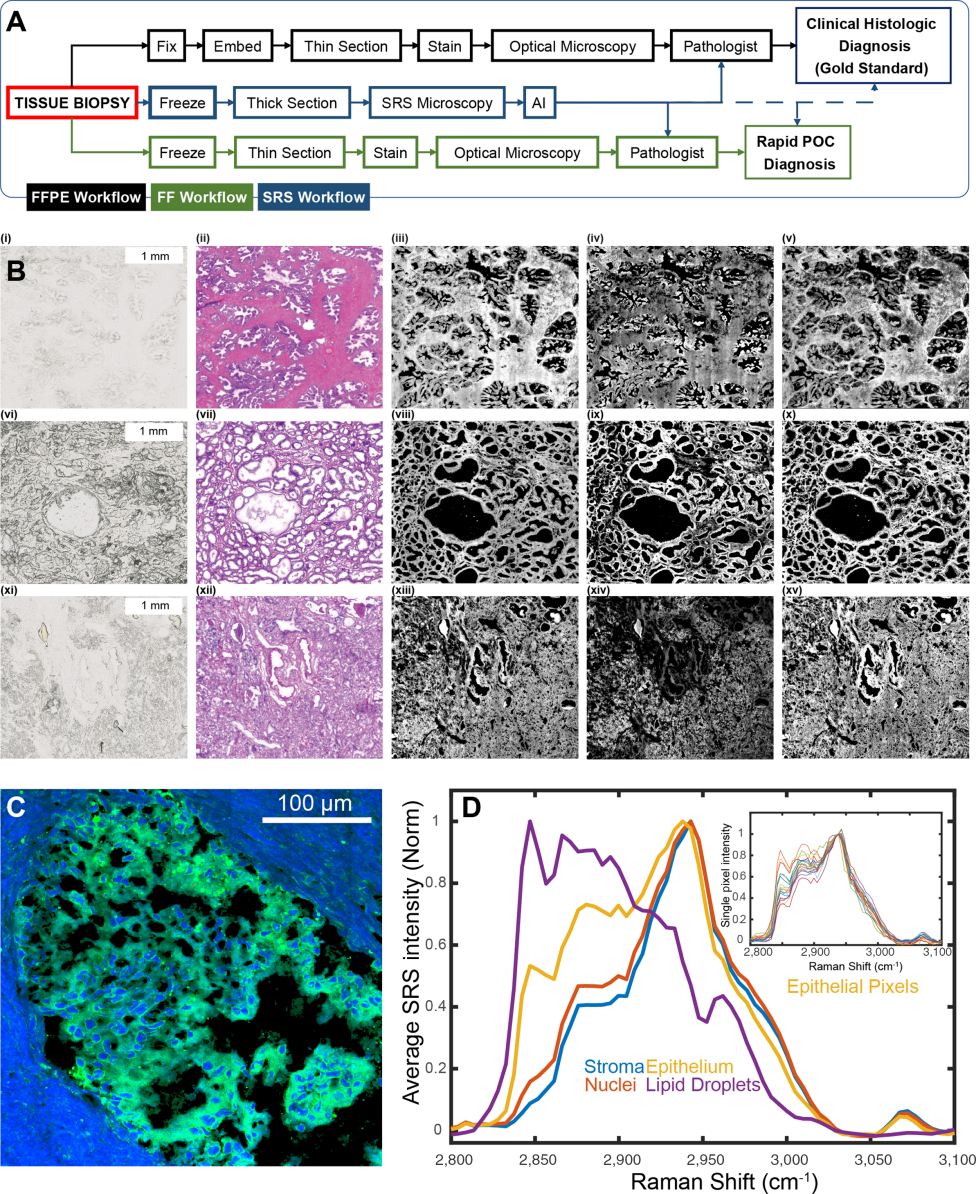
Overview of the experiment and results presented in this article. **A,** Comparison of the presented SRS histopathologic workflow with standard workflows (POC, point of care). **B** (i)–(v), results for benign tissue. (i) Brightfield image of unstained tissue; (ii) brightfield image of H&E-stained adjacent section; (iii) SRS image at 2933/cm^−1^ showing overall tissue composition; (iv) SRS image at 2847/cm^−1^ highlighting epithelium; and (v) SRS image at 2979/cm^−1^ highlighting stroma and blood vessels. **B** (vi)–(x), results for low-grade cancer (GS = 3). (vi) Brightfield image of unstained tissue; (vii) brightfield image of H&E-stained adjacent section; (viii) SRS image at 2933/cm^−1^, (ix) SRS image at 2847/cm^−1^, and (x) SRS image at 2979/cm^−1^. **B** (xi)–(xv), Results for high-grade cancer (GS = 4). **B,** (xi) Brightfield image of unstained tissue; (xii) brightfield image of H&E-stained adjacent section; (xiii) SRS image at 2933/cm^−1^; (xiv) SRS image at 2847/cm^−1^; and (xv) SRS image at 2979/cm^−1^. **C,** Two-color SRS images (as described in the text) of a zoomed in region of a gland. **D,** Average max normalized SRS spectra of stroma, nuclei, epithelium, and LDs (color coded). Inset, spectral heterogeneity of epithelial cells shown in C.

**FIGURE 2:**
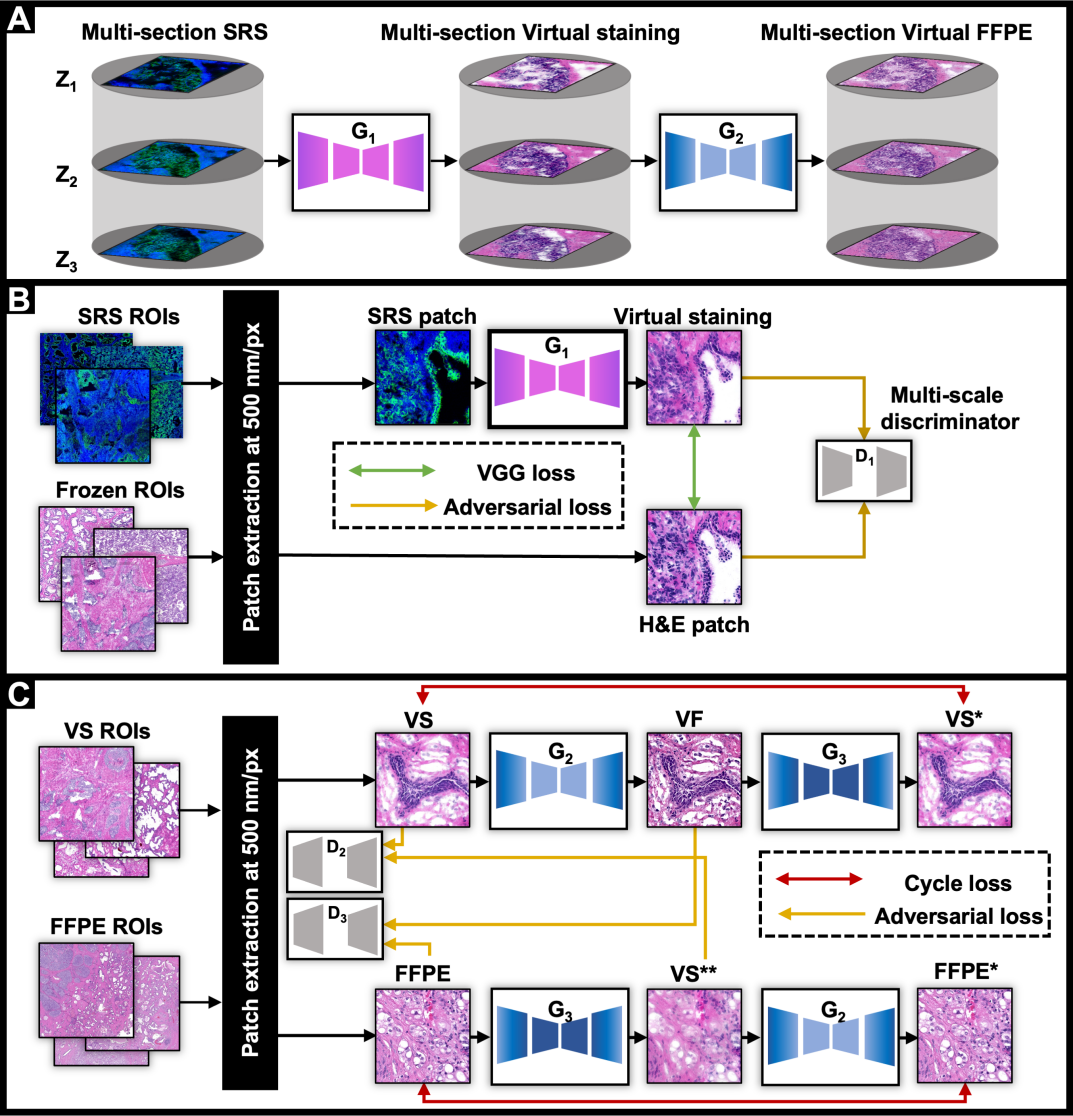
Recorded data and predicted images in workflow. **A,** An overview of the proposed approach, acquiring multidepth data from surgical resections and using DL frameworks to generate H&E-stained images from SRSM data from FF tissue (G1) and FFPE-equivalent images (G2). **B,** Concept diagram of the staining model (G1) that converts SRSM data to H&E images obtained from FF tissues (virtual FF). **C,** Concept diagram for the model (G2) to convert virtual staining images to FFPE-like images (virtual FFPE). D_1_, D_2_, and D_3_ are the discriminators. G3 is generating virtual FF samples given FFPE samples, which is part of the training algorithm.

We present an overview of our approach and results in Fig. 1. In Fig. 2, we introduce the DL framework, which converts label-free SRSM images of FF samples to FFPE-like conventional H&E, useful for clinical activities. As shown in Fig. 2A, this framework includes two generators: G1 and G2. The first model, G1, is the virtual staining model that transforms SRS images into H&E images of FF tissues. As can be seen in Fig. 2B, G1 follows GAN formulation where its parameters are optimized by minimizing mean square error (MSE) loss and adversarial loss. The second model, G2, is responsible to enhance the morphology of those virtual staining images and turn them to FFPE-like samples. Because the FF and FFPE images are not paired, we utilize cycle-GAN (44) to train G2 based on an unpaired dataset (Fig. 2C). G3 maps images from the FFPE domain to FF as part of cycle-GAN methodology. For calculating the adversarial loss, we use multi-scale discriminators where there are two discriminators that have an identical layout and we apply them to different image scale similar to the previous study (34). Briefly, one of the discriminators operates on the full resolution images whereas the other one operates on the downsampled images by factor of 2. In addition, both generators follow a modified U-Net architecture that has been successfully implemented previously (45).

### Virtual Staining

We trained G1 in two phases. In phase I, we minimized the MSE loss function between the G1’s output and the real FF H&E ground truth for 50,000 iterations. However, as can be seen in Supplementary Fig. S1, the results using MSE loss function is blurry and overly smooth, which has been reported previously (45, 46). Thus, in phase II, we retrain G1 using the pretrained model in phase I and optimizing weights using a GAN formulation (Fig. 2B) for 200,000 more iterations using batch size of 9:



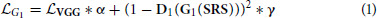



Where 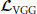 is perceptual loss, similar to the previous study (45) that is calculated by MSE of feature maps produced by pretrained VGG-19 model (47). α and γ in Eq. (1) are regularization terms to stabilize the training, which we set them to 0.01 and 0.005, respectively.

The discriminator loss is defined as:



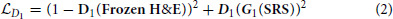



### Virtual FFPE

We train virtual FFPE model using a cycle-GAN framework similar to the previous study (44). As can be seen in Fig. 2C, there are two loss functions: cycle loss and adversarial loss. Cycle loss is defined as below:



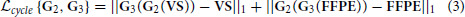



Where VS is the virtual staining image resulting **G**_1_. The adversarial loss for translating VS to FFPE is defined as:



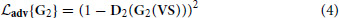





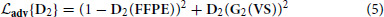



Similarly, for mapping from FFPE to VS, 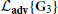 and 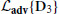 are calculated in the same way. The overall objective function is defined as below:



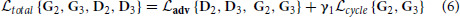



Where 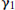 is a regularization term and set to 10. We train virtual FFPE framework for 100,000 iterations and batch size of 6.

### Pathologist Review

To evaluate the virtual staining results, we created a comprehensive survey and sent it to five urologic pathologists. The purpose of this survey was to determine the effect of virtual staining on the quality of diagnosis. We asked three key questions in the survey for each image:

- What is the quality of this FF section?
- What is the quality of staining?
- What is the predominant Gleason pattern?

The first question measures the quality of FF sections using virtual staining and actual staining. The idea is to make sure our framework does not introduce additional artifacts related to FF samples. The second question evaluates the quality of staining for virtual stain and real stain images. Finally, the third question measures pathologists’ concordance rate in assigning Gleason pattern for real and virtual stain images. We evaluated the interobserver agreement using Fleiss’ kappa statistic, which is a common statistical approach for analyzing interobserver variability. Fleiss’ kappa takes into whether an agreement is by chance or not. Fleiss’ kappa value of 0 demonstrates agreement occurring by chance and a value of 1 shows perfect agreement. In addition, we calculated the minimum number of samples required for the survey using previous study (48) where the survey was expected to detect 0.15 difference in kappa for 90% confidence intreval for grading of virtual stain and actual H&E stain images. As a result, the survey included 35 virtual staining images and 35 FF H&E images from the same or adjacent section. The field of view of the images was in range of 1 to 20 mm^2^ and pathologists had access to navigation tools such as zoom in. This field of view strikes a balance between evaluating the entire slide, which may result in variable weighting of different regions and does not allow consistency in the region on which a pathologist may focus, and evaluating patch images, which do not capture the complexity and heterogeneity of prostate cancer adequately.

### Algorithm Implementation

The framework is implemented in PyTorch 1.3, CUDA 10.1, and Python 3.7.1 (IPython, RRID:SCR_001658) and computations are performed on a three NVIDIA GeForce RTX 2080 Ti 12 GB GPUs and Intel(R) Xeon(R) Silver 4216 CPU @ 2.10 GHz. All of the generative and discriminative models are initialized to random weights. Adam (49) was used to optimize the parameters of models with an initial learning rate of 10^−4^ and they are multiplied by 0.96 after every 1,000 iterations. For data augmentation, we randomly selected a region of 384 × 384 from the 500 × 500 patches at each iteration and we applied random affine transformation to the resulting images.

### Statistical Analysis

The ANOVA and *t* test results presented in this article were performed with Matlab R2018b (MathWorks).

### Data Availability

The data generated in this study are available upon request from the corresponding author. The codes for generating the virtual FF and FFPE images can be downloaded from https://codeocean.com/capsule/3060667/tree/v1.



## Results

The comparison of the proposed workflow with current processes is presented in Fig. 1A. The proposed SRS-based histopathologic workflow (middle) includes two major changes—first, the use of relatively thick tissue sections that are easier to cut than thin sections. Second, dispensing with the need for staining by using AI. Notably, this workflow seeks to generate both FF and FFPE images of thin sections from thick sections, providing the pathologist with high-quality images. To illustrate the opportunities and challenges in devising this workflow, we examine images from different contrast mechanisms first. As with all tissues, unstained histologic sections have very little contrast in brightfield microscopy [Fig. 1B (i), (vi), and (xi)]. Stains highlight specific features, for example in Fig. 1B (ii), (vii), and (xii) highlight glandular and stromal patterns that become appreciable due to the contrast between the nuclei largely found in epithelial cells (staining blue with hematoxylin) nestled in the protein-rich stroma and extracellular matrix (staining pink with eosin). From H&E-stained images, pathologists identify different morphologic patterns of epithelial cells (Gleason grades) that are known to be correlated with prostate cancer severity and outcome. In SRSM, chemically-sensitive images from protein, nucleic acid, and lipid composition can be directly generated without any dyes or stains. Figure 1B (iii), (viii), and (xiii) show the overall tissue composition at 2,933 cm^−1^, Fig. 1B (iv), (ix), and (xiv) highlight the epithelium at 2,847 cm^−1^, and Fig. 1B (v), (x), and (xv) highlight the stroma at 2,979 cm^−1^. Conventionally, dual-frequency SRS images are presented with *SRS_2933_-SRS_2847_* as false color blue images (mimicking DAPI stains of nuclei) and *SRS_2847_* as false color green images mimicking actin stains, as presented for a zoomed-in section of a gland in Fig. 1C, highlighting the nuclei and stroma (blue) and cytoplasm of epithelial cells (green). While using SRSM data to produce H&E images has been demonstrated, generating realistic staining patterns for a wide variety of cells is difficult due to subtle morphologic and contextual differences. For example, nuclei of basal epithelial cells are morphologically distinct from mature epithelial cells. There are also spectral differences across voxels that arise from underlying biological and functional differences, biochemical heterogeneity even in physiologically-identical cell types, sampling differences and noise. In Fig. 1D, we show the average normalized spectra from tissue compartments. First, differences in these spectra illustrate the powerful potential of spectroscopic imaging to segment cells and subcellular domains. Second, the heterogeneity of composition even in this small set from a single sample (inset figure, epithelial pixels) presents the challenge of simply using spectral markers for segmentation. Our AI approach sought to harness spectral differences between cell types as well as overcome within cell-type variations using spatial-spectral methods with DL.

The first task was to choose specific SRSM frequency bands for analyses. We determined the importance of bands by considering their biological significance and their ability to reconstruct the whole spectra using a regression model, reporting frequencies used in Supplementary Fig. S2. To convert these multiband discrete frequency SRS images to clinical images, we developed two DL frameworks (Fig. 2A) that include two convolutional neural networks generator models: G1 and G2. G1 transforms SRS images into H&E images of FF processed tissues. As shown in Fig. 2B, G1 follows the GAN formulation wherein its parameters are optimized by minimizing MSE and adversarial loss, as detailed in the Materials and Methods section. The second model (G2) enhances contrast to simulate the results of tissue processing and, additionally, mitigates FF sample artifacts to convert the morphology of those virtual FF-stained images to archival-quality FFPE-like ones. An additional challenge in developing these models was that FF and FFPE images cannot be acquired from the same sample, that is, are not paired. We thus utilized cycle-GAN to train G2 based on an unpaired dataset (Fig. 2C).

For creating the training and test datasets for G1, we imaged ROIs from 70 FF prostate samples. For training, we used ROIs from 31 (25 stained using the same sections and six stained using adjacent sections) samples resulting 5,023 patches of 500 pixels × 500 pixels. The remaining 39 samples were used for testing. This dual-sample type strategy allows a highly precise “matched” set that provides the ability to precisely compare stained and generated images whereas adjacent sections provide a slight diversity that is expected to make training more robust. The SRS and FF H&E images must be correlated for G1 training. Because staining introduces tissue deformation and some stained images are from adjacent sections, perfect pixel level registration is not straightforward. One option is to develop sophisticated alignment algorithms with deformation, translation, and rotation models. However, it is unclear whether that approach can provide precise alignment universally. Instead, we macroscopically align the SRS and H&E images using control points over the whole section while understanding that there will be some noise in the correlations that will result from small misalignments. Matched patches were used as inputs for training using 200,000 iterations (more details in Materials and Methods).

**FIGURE 3:**
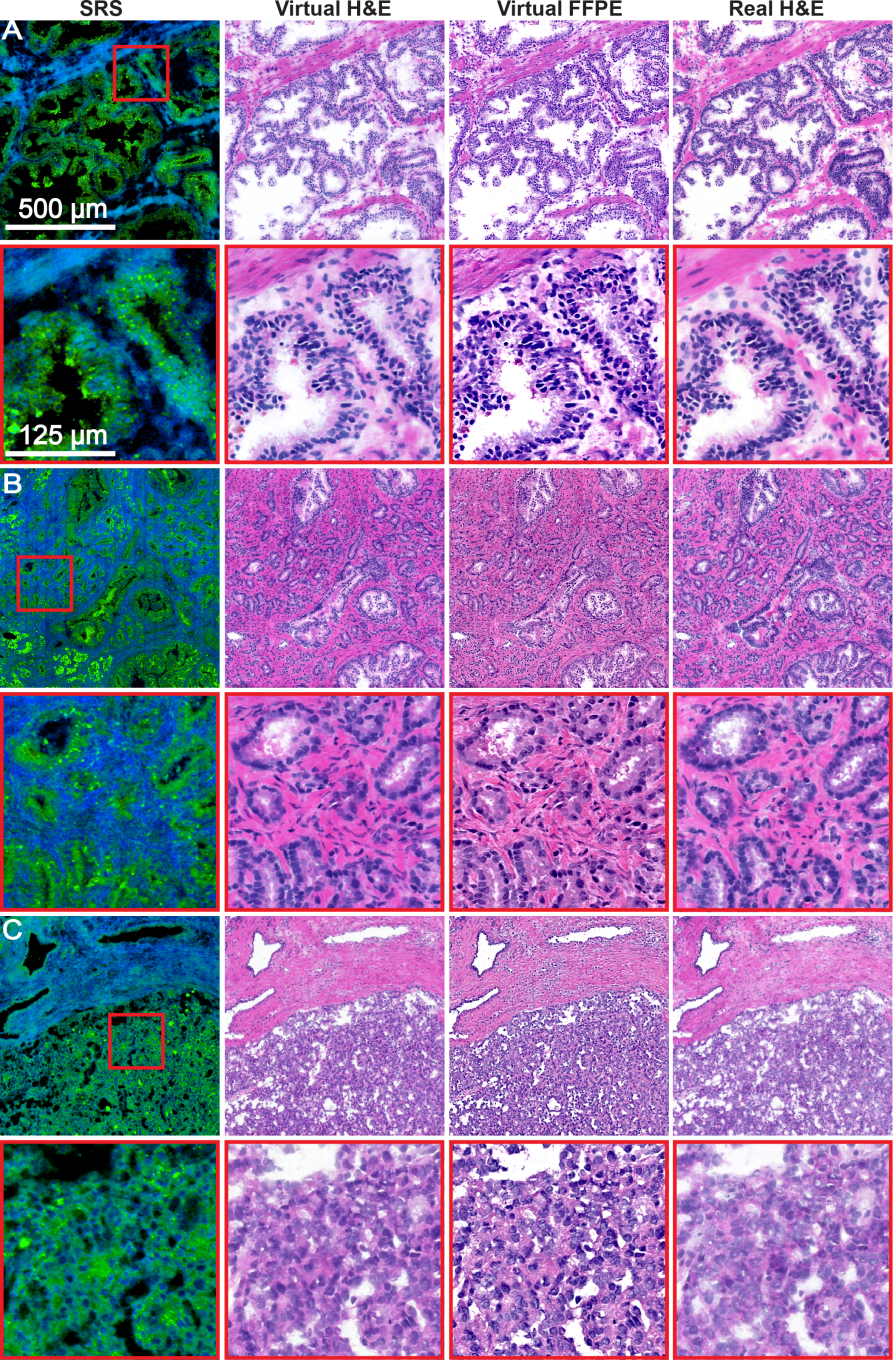
Comparison of virtually stained images from SRSM and stained images using optical microscopy for FF tissue. **A,** Benign. **B,** Low-grade cancer. **C,** High-grade cancer. From left to right, we show SRS image, virtual-stain image, virtual FFPE image, and real H&E image.

The framework's performance is evaluated by using SRS images from patients that were not a part of the training and testing sets (blinded external validation). Figure 3 illustrates the results for three major representative pathologies. From left to right, we show chemical images, the generated FF H&E image, virtual FFPE H&E image, and corresponding stained images. Two-color SRS images are presented as described in Fig. 1C. Figure 3A compares virtual and H&E images for normal tissue with open glands in stroma with polarized epithelial cells, which are well reproduced. Figure 3B shows an example of a low-grade cancer (Gleason grade 3 and below), in which crowded, filled-in but identifiable glands, enlarged epithelial nuclei and dense stroma can be identified. Figure 3C shows an example of a high-grade cancer (Gleason grade 4 and 5) in which the lack gland formation and heterogeneity of nuclear signals can be readily appreciated. Examination of all samples in our dataset showed similar performance, with key hallmarks of disease severity being appreciable. Examples are shown in Supplementary Fig. S3. Notably, while the DL framework is able to capture important histopathologic features like glands, epithelial cells’ nuclear details and stromal tissue, there are also some instances of inconsistencies of data between ground truth and virtual stain. For example, the level of inflammation in stroma in not in complete agreement with the H&E-stained ground truth (Supplementary Fig. S4). These differences likely arise from the relatively low abundance of immune cells and will improve with more data. Because our focus here was to reproduce key diagnostic features used in clinical pathology, we did not attempt to further improve the model.

To evaluate the performance of our workflow in terms of clinical utility, a panel of five pathologists was enrolled to conduct an evaluation of the results. The pathologists are experienced pathologists with expertise in urologic pathology at Mayo Clinic. The evaluation of our images was conducted using an online survey, with 35 examples each of virtual and stained images for FF sections. The images were randomized in the order of presented images for each pathologist. The first two tasks were to assign a score to each image based on the quality of the image in the “prepared” slide and stain quality for each image. The score ranged from 1 (low quality) to 5 (high quality). Because there cannot be an absolute scale to measure, we evaluated the performance of each pathologist within their own scores’ distributions for the same set of 70 images. As summarized in Table 1, pathologists assigned relatively similar scores to both virtual and real images, without statistical differences in the ratings. In general, the pathologists observed a greater consistency in “slide” preparation quality and slightly preferred the virtual images though we emphasize the results are not statistically different. This may be due to the uniformity of image quality that can be achieved by directly measuring tissue chemistry rather than the artifacts introduced sometimes by dyes. In terms of using the images by assessing stain quality, pathologists slightly preferred physical stains though we emphasize the results are not statistically different. This may be due to the lower attention we paid to reproducing stromal features and texture that may make the images appear a little washed out. Regardless, the similarity of the assessment of quality indicates that virtual stain images are comparable. For the third task, we calculated the utility of using the virtual images for diagnostic applications by calculating interobserver agreements using Fleiss’ kappa to capture both accuracy and disagreements that may arise. We evaluated the performance of pathologists in distinguishing benign from cancerous samples first, as a simulation for assessing margins, and then assessed the performance in assigning tumor grade. As shown in Table 2, for grading the samples, Fleiss’ kappa value for virtual stain and real stain images is 0.496 and 0.484, respectively. This indicates the same level for recognition of disease. For grading prostate cancer, similarly, it is 0.491 and 0.473, respectively. It is important to note that a kappa value 0 demonstrates agreement by chance and 0.01–0.20 as none to slight, 0.21–0.40 as fair, 0.41– 0.60 as moderate, 0.61–0.80 as substantial, and 0.81–1.00 as almost perfect agreement (50). Thus, our findings suggest that pathologists’ agreement for virtual and real stain images is moderate. This result is in concordance with reported concordance rates of Gleason scoring (51) and is slightly lower (0.55 vs. 0.49) as anticipated for difficulties grading FF samples.

**TABLE 1:** Pathologists review from the survey for assigning section and stain quality score, where we report mean ± SD of the scores that have been assessed by pathologists to each domain

#ID	#1	#2	#3	#4	#5	Average
Assigning section quality by pathologists						
Virtual stain	3.43 ± 1.33	4.31 ± 1.02	3.43 ± 0.85	2.91 ± 0.92	3.66 ± 1.30	3.56 ± 1.18
Stain	3.80 ± 1.16	4.20 ± 1.02	3.20 ± 0.99	2.71 ± 1.02	3.40 ± 1.26	3.47 ± 1.20
Assigning stain quality by pathologists						
Virtual stain	3.00 ± 1.35	4.29 ± 0.86	3.40 ± 0.85	2.94 ± 0.72	3.94 ± 1.08	3.52 ± 1.12
Stain	3.46 ± 1.15	4.34 ± 0.59	3.26 ± 0.92	2.71 ± 0.71	3.89 ± 0.87	3.54 ± 1.02

NOTE: Scores interval is from 1 to 5, where 1 low-quality samples and 5 being high-quality samples.

**TABLE 2:** Pathologists review results from the survey for calculation of interobserver agreement for each domain using Fleiss’ kappa for 95% confidence interval

	Grading
Virtual stain	0.491 (0.416–0.566)
Stain	0.473 (0.397–0.549)

**FIGURE 4:**
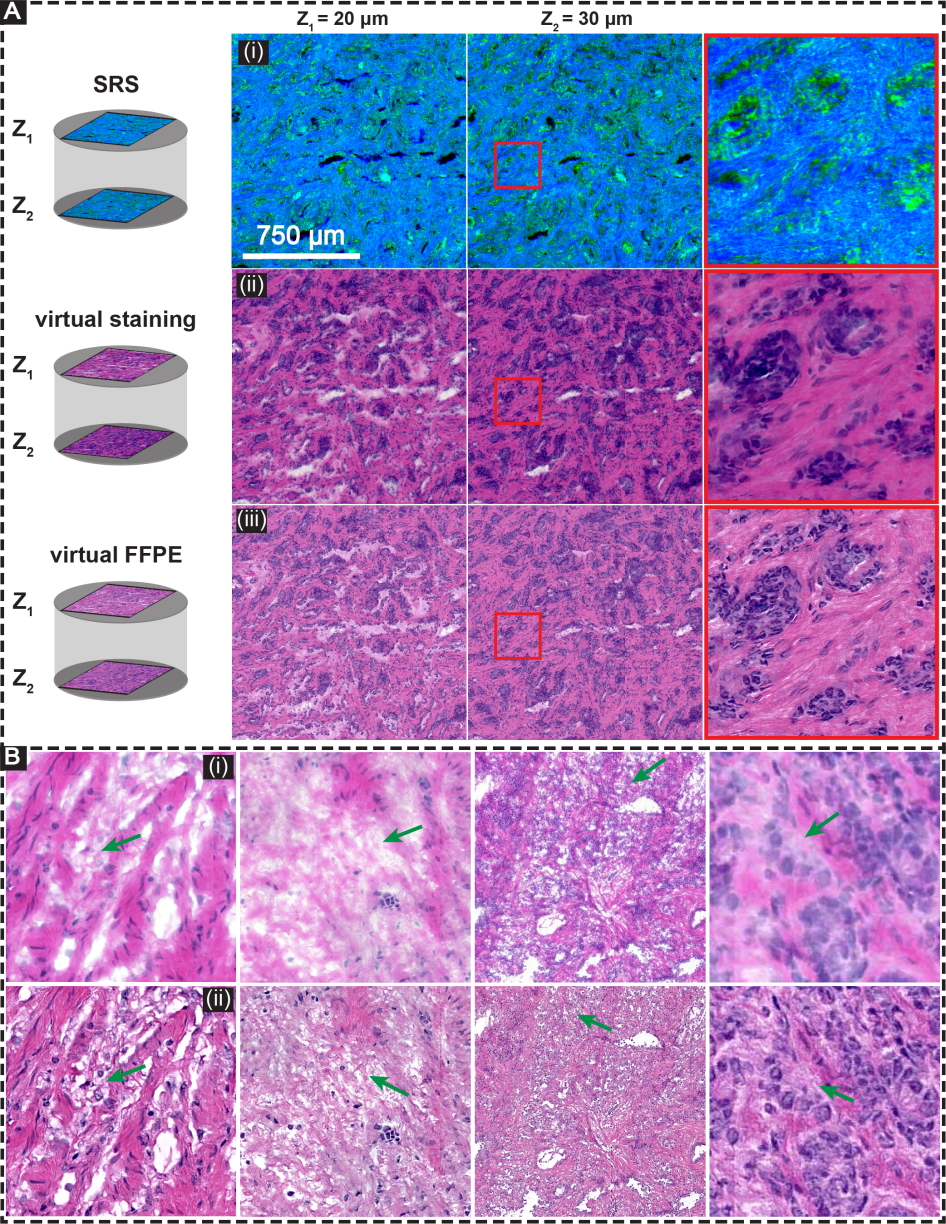
Virtual FFPE. **A,** Multisection SRS (i), are fed into G1 to generate multi-section virtual staining image (ii), which is then input into G2 to produce multisection virtual FFPE images (iii). **B,** Examples of mitigating freezing artifacts that exist in FF samples (i), using G2 network to generate virtual FFPE images (ii). From left to right, we demonstrate staining, freezing, knife cut, and blurring artifacts.

Obtaining thin slices from FF tissue requires skill, experience and can lead to artifacts that may confound accurate diagnoses. The optical sectioning capability of SRS can mitigate such artifacts by confocal imaging of thicker tissue. Here, we seek to show this potential by imaging relatively thick sections that are easier to cut and less prone to artifacts (∼50–100 µm) while seeking to obtain thin section quality images. We image these sections across planes at different depths and computationally generate the virtual stain images by applying the DL framework developed above. Figure 4A (i) shows representative SRS data at two different depths (20 and 30 µm) of the thick low-grade prostate cancer section. Even in this thickness, a diversity in pathology and glandular structures can be seen in corresponding multi-section virtual stain image [Fig. 4A (ii)]. Importantly, multi-section virtual staining allows for a more thorough examination of the sample in limited time settings such as intraoperative pathology. One of the key benefits of multi-section virtual staining is that it reduces the chance of missing tumor in the histologic images. This is because tumor cells do not always exist in every section of a tissue sample, and traditional staining methods may only examine a limited number of sections. With multi-section virtual staining, a larger number of sections can be examined, decreasing the likelihood of overlooking cancerous regions. In addition, this approach can also provide a more detailed and accurate understanding of the tumor's size, location, and cellular characteristics, which is important for diagnosis and treatment planning. To this end, in Supplementary Fig. S5, we show representative multi-section virtual stain images at three different depths of the thick section. At a depth Z_1_ = 0 µm, there is no tumor that can be identified. By looking at virtual stain images at Z_2_ = 20 µm and Z_3_ = 40 µm, the cancerous regions are clearly apparent. It is well known that FF sections lack sufficient morphologic quality for definitive diagnosis; hence, pathologists conduct gold standard diagnoses by preparing FFPE sections of the same sample even if FF histopathology is conducted. To improve the morphologic information and staining contrast of FF images (44), we developed a DL framework (G2) to generate FFPE-like images (virtual FFPE) from virtual FF images. In Fig. 4A (iii), we demonstrate the performance of G2 for generating virtual FFPE images given virtual stain images. We apply the model to various depths of sample separately to create multi-section virtual FFPE images. To train G2, we selected 25 samples from virtual stain images and 25 samples from real FFPE samples. For training, 4,608 patches of 500 × 500 were created. It is important to note that after the training phase, the model recovers correct FFPE morphology and contrast without the need for additional information.

In Fig. 4B, we show four examples of FF artifacts including staining, freezing, knife cut, and blurring artifacts, which have been mitigated using virtual FFPE model. Our findings indicate that utilizing a virtual FFPE network improves the appearance of FF histologic images by increasing the clarity of morphologic features, providing more detailed structure, and enhancing color contrast, which aligns with previous study (44). Notably, our method of creating volumetric data is distinctive as it eliminates the need for sectioning, staining, and preparing FFPE samples separately. Instead, it uses a SRS system to image thick FF samples, followed by AI to create multi-section virtual FFPE images without the need for slicing the samples. This makes our approach more efficient and less time-consuming compared with conventional gold standard histopathology. It is important to note that because signal localization depends only on the focal spot and there still significant transmission through these thicknesses, we do not expect any physical reason for a degradation in image quality compared with the thin section results reported previously. Collectively, these results demonstrate both virtual stains and optical sectioning capability in our workflow that is geared to be simpler in sample processing and can likely result in fewer artifacts, but the utility of our results needs to be evaluated in a clinical context.

**FIGURE 5:**
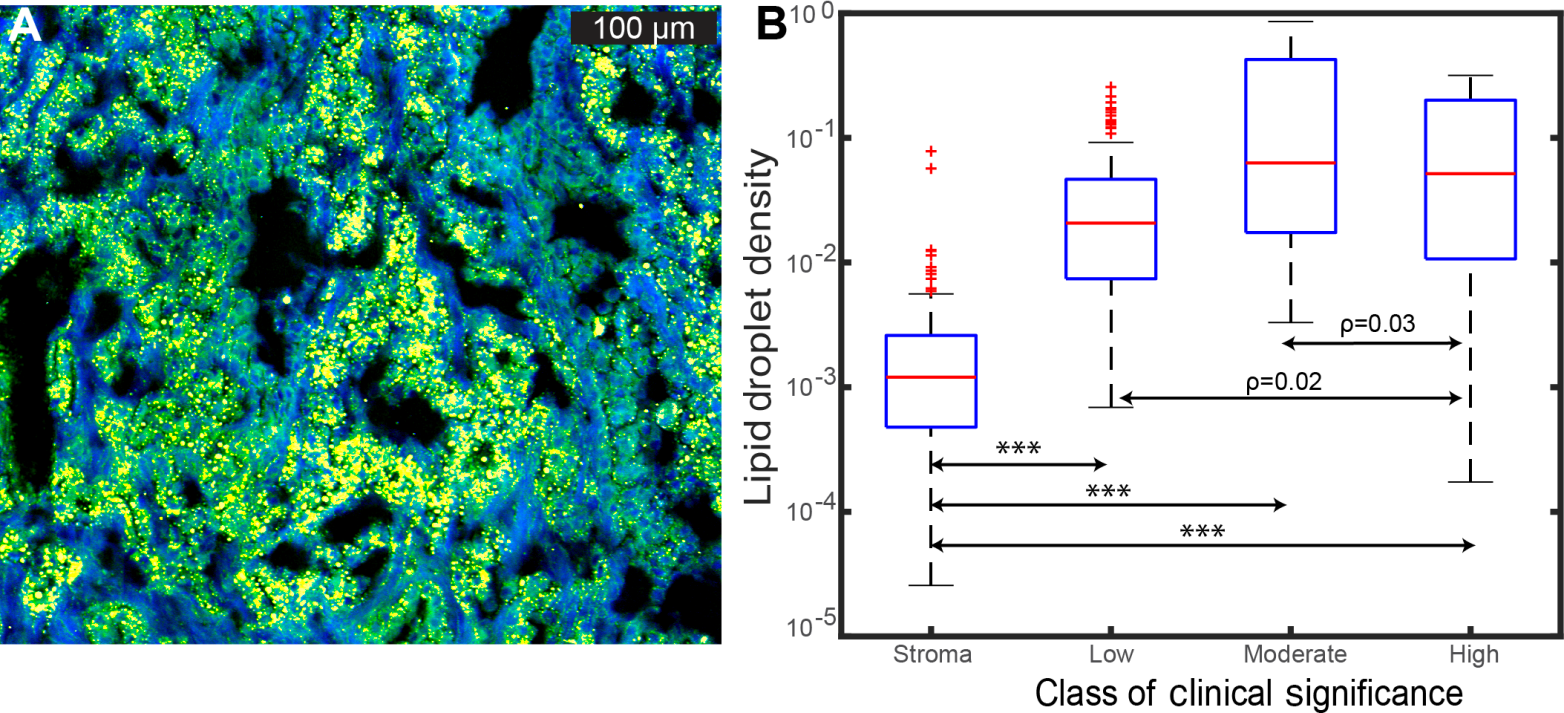
LD density in prostate cancer. **A,** Representative SRS image of a high-grade cancer gland (GS = 5) showing heterogeneous expression of LDs (yellow pixels). **B,** Box plot showing LD density (log scale) for the different classes of clinical significance as described in the text. The two highest *P* values of the pairwise unpaired *t* test as described in the text is shown, the rest are all highly significant (*P* ≤ 1E-7) and are marked by ***.

While the previous sections allow condensation of the workflow, SRSM offers an additional advantage. The use of FF tissue sections and minimal processing preserves the native lipid composition of tissues and enables correlative study of lipid droplet (LD) expression with grades of prostate cancer. Upregulation of *de novo* lipid biosynthesis leading to accumulation of lipids in the form of droplets has been identified as a potential biomarker for prostate cancer aggressiveness (52, 53). To explore the prognostic value of LD expression in prostate cancer, we analyzed the SRS images from tissues of a subset of 33 patients divided into three major categories of clinical relevance—of low [Benign through low Gleason patterns (GP), GP < 3], of moderate (GP = 3) and high clinical concern (GP = 4 and 5). Because prostate cancer is a highly multi-focal cancer with multiple Gleason patterns often present in close association, analyses were performed at the level of individual glands or contiguous tumor areas which were unambiguously assignable to a pattern. To evaluate the changes in lipid expression in the stromal cells (fibroblasts, smooth muscles, etc.), segments of stroma adjacent to glands were chosen with no infiltrating cancer cells, inflammation and other abnormalities. A total of 96 gland patterns of low concern, 56 of moderate, and 61 of high concern. Furthermore, 119 ROIs in the stroma were annotated for statistical analyses presented below. From the average spectra of LDs presented in Fig. 1D, we generated a threshold value of the ratio: *SRS* (2,847 cm^−^^1^)/*SRS* (2,933 cm^−^^1^) for quantifying the presence of LDs. Finally, the segmented images were used to calculate LD density. Analogous to the histomorphic heterogeneity of prostate cancer, the LD density also shows large overall intraclass heterogeneity (i.e., same pattern in different regions of the tissue and across different patients) for patterns of moderate and high concerns (interquartile range/mean value of 1.7559 and 1.7562, respectively) as compared with that of patterns of low clinical concern (0.9063). Figure 5A is a representative example of a cancerous gland showing heterogeneous LD expression, apparent as roughly circular droplets in a GP = 5 adenocarcinoma. Figure 5B demonstrates the observed LD density (log scale) for the different classes of clinical significance described above. One-way ANOVA (*F* = 129.62, ρ = 2.19011e-55) followed by *post hoc* pairwise unpaired *t* test between the groups reveal statistically significant differences (at 5% significance level) in mean LD densities between all the groups (ANOVA and *t* test results detailed in Supplementary Tables S1 and S2). Interestingly, the cancer glands that lead to moderate clinical concern (GP = 3) show the highest levels of LD distribution followed by those of high clinical concern patterns (GP 4 and 5). In contrast, glands of low clinical concern and stroma showed negligible presence of LDs. Overall, this result demonstrates that although lipid metabolism is upregulated in clinically significant prostate cancer, as evidenced by the higher LD density, there is significant heterogeneity at the level of individual glands and the correlation with cancer patterns is not monotonic. Further studies are needed to evaluate the effects of other confounding factors. This study could prove foundational for these future studies as it allows the simultaneous observation of both lipid patterns (which are only present in FF tissue) and gold standard pathology images (which are obtained after FFPE processing and loss of lipids).

## Discussion

Our proposed method simplifies several steps in pathology—first, we seek to reduce time from surgical or biopsy tissue acquisition to H&E images from days to minutes by eliminating numerous steps in contemporary clinical sample processing and staining. To our knowledge, the goal of this study to generate archival-quality FFPE images rapidly from FF processed tissue, is unique. While we have focused on clinical, human specimens, the same benefits can be expected in research studies and studies involving animal tissue. Reduction of processing tasks and effort could be especially beneficial for accelerating research as well as preserving the tissue for further tests. The ability to generate FFPE images can also be translated to *in vivo* measurements. Given the complexity that would be involved in microscopically imaging the prostate, we did not attempt that translation in this study. However, other more accessible organs and animal models could benefit from such an extension of this study. Second, we utilize thick sections (∼100 µm) that make the FF pathology process easier and faster. There is an unmet need to accelerate the speed and quality of FF processing. Preparing thin sections (<10 µm) is laborious and needs skill under the time constraints where FF processing is most useful. FF processing can also often introduce artifacts due to cell swelling, ice crystal development, folded and lost tissue, and uneven staining, resulting in poor morphologic detail. Current methods are also not conducive to cutting thin sections from certain types of tissue, for example, breast tissue containing fat. These issues have resulted in an underutilization of intraoperative pathologic assessment to the detriment of patients in a variety of cancers (54). Because our approach can be translated to other tissues and other venues, further, it can address these drawbacks in FF processing.

Third, we use the optical sectioning capability of SRSM to generate more than one image (at different depths) per thick section and provide more data for interpretation. The ability to assess tissue at greater depths has the potential to increase the sensitivity of detecting positive margins as well as for other assessments such as lymph node metastases in sentinel lymph nodes. Finally, we seek to connect the FF and FFPE domains using AI from SRSM images. In principle, chemical imaging can provide cellular and disease recognition (30) that can lead directly to diagnostic information (as indicated by the dashed lines in Fig. 1A). However, our goal in this study is limited to generating and evaluating images for use by pathologists. In general, good agreement was found between the diagnoses on traditional histologic sections and those made on the virtual sections. While utilizing adjacent sections for some evaluations in this study might have a minor impact on the scoring, the 5-µm difference between the sections is relatively small and should not significantly affect the results. It is notable that our samples were prepared by a highly qualified and experienced team in a controlled setting, thereby minimizing artifacts. In a time-sensitive clinical preparation of FF sections or with less experienced personnel, the utility of suppressing artifacts (55) in virtual stains can be even more valuable. Because the AI-powered imaging technology can record data without specialized reagents or the need for expert, multi-step manual processes, it can be translated from major academic medical centers to community centers. Thus, high-quality pathology images can be obtained rapidly even in settings with fewer human resources.

In this study, we present a DL framework that facilitates faster pathologic evaluation of FF samples. Without any staining and additional processing of the tissues, our framework computationally converts multi-section SRS images of thick samples into H&E images and further into the multi-section virtual FFPE domain. Because of the well-represented morphologic details and color schemes of virtual FFPE images, the current study facilitates pathologists’ workflow in evaluating FF samples for screening as well as reducing the potential for diagnostic errors as discovered previously (44). To further evaluate the framework, a large-scale clinical study is needed to compare it with conventional pathology, which will be important to understand the potential failure cases of our framework. These cases can then be included to provide feedback to the models by penalizing such failures and subsequent retraining for further optimizing and generalizing the output. Although the central focus of this study is on SRS imaging, other label-free imaging modalities and contrast mechanisms such as infrared imaging, polarization imaging, and autofluorescence imaging can use similar approach for histologic analysis and data reconstruction. The same approach can also be applied to any other types of tissues or for generating different types of stains. Currently, the pixel dwell time of SRS microscopy is still higher than commercial slide scanner systems. However, with improvements of instrumentation of SRS microscopes and incorporating AI-based computational tools, it would be possible to have “real-time” analysis, thereby reducing (16) the time needed for sectioning, staining, and evaluation. SRSM also has several key advantages over infrared absorption based chemical imaging such as Fourier transform infrared (25) and discrete frequency (56) infrared imaging; including the ability to image wet tissues, higher axial and lateral diffraction-limited spatial resolutions at multiple depths. However, the molecular sensitivity and spectral range of SRSM is comparatively limited. As DL approaches appreciate higher spatial resolution (45), they can be effective with smaller spectral differences and SRSM data are ideally suitable to a DL-based workflow. In comparison with non–DL-based transformation methods, like the method published by Sarri and colleagues (18), it is more robust to noise or stain variations, generates more realistic stained images and after the training phase is fully automated. On the other hand, as with any DL-based technique, training model involves large and heterogeneous dataset, optimization of large number of hyperparameters and careful validation by expert pathologists.

A current drawback of our proposed workflow is its increased complexity and cost-effectiveness compared with traditional pathology. We emphasize that the integration of SRSM and DL processes in pathology laboratories does not seek to replace staining from a cost consideration as a standard diagnostic workflow. In fact, using only light, the tissue remains available for traditional staining. Nor do we expect the universal use of this workflow. However, for rapid assessment of tissue in intraoperative settings, to triage from amongst many samples or for research purposes, obtaining archival quality images in a much-reduced time period can be highly beneficial and routinely afford more information that takes time to obtain. As with most technological advancements, continued development can make this approach more practical and affordable. Regardless, it offers a unique cost-benefit alternative to augment pathology. For example, the proposed approach could enable intraoperative assessment of tissue in community settings that conduct the majority of cancer surgeries but lack the resources of major academic medical centers to provide rapid pathologic assessments. The results described here should provide an additional avenue for development and use case for this technology, providing an alternative for specific use cases where it may be advantageous to implement shortened workflows.

Because our approach only uses light, the tissue can be processed for traditional pathology or other workflows after imaging. More detailed chemical analysis can be conducted using SRSM itself as well. It is well known that cancer cells have altered metabolic pathways to sustain the proliferative growth of tumor. Among others, *de novo* lipogenesis and altered lipolysis are increasingly getting recognized as hallmarks of many forms of cancer including prostate cancer (57). This can be manifested in accumulation fatty acids in LDs. In addition, cholesteryl esters are reported to have a key role to play in disease progression in prostate cancer and can be stored in the form of LDs (57, 58)*.* However, there is little consensus on the correlation between cancer severity (Gleason score) and LD accumulation. A previous study in a mouse model (53) demonstrated altered LD density and correlation with the Gleason pattern, two recent studies with human prostate cancer tissue showed contradictory results. While the hyperspectral SRS imaging study by Chen and colleagues (59) found no significant difference between low-grade (GS ≤ 6) and high-grade (GS ≥ 6) prostate cancer, a more recent study (21) found statistically significant difference between all except Gleason patterns 4 and 5. Our results show that while there is a large statistically significant difference between benign and cancerous glands, there is a smaller difference between low-grade and high-grade cancer (ρ = 0.03), with low grades seemingly having more LD expression than high grade. However, it is not entirely clear whether this originates from finite sampling (because the significance level is relatively low; Supplementary Table S2) or will be broadly diagnostic. The workflow proposed here can be used for rapid assessment of tissues and addressing this question. Another important observation is the heterogeneity of LDs in the same patient (particularly for low grade cancer; Supplementary Fig. S6) between adjacent glands, suggesting that Gleason grading and the metabolic signature may not be uniformly distributed. Measures of heterogeneity in expression and the significance of this heterogeneity emerges as an interesting avenue to explore. Given the variability in morphometric assignment, a better understanding of LD accumulation and correlation with key enzymes and regulatory proteins of lipid metabolism is necessary. This further represents a merit of our workflow with FF samples because it is fully compatible with IHC-based imaging. Finally, because the imaging modality and the machine learning algorithms are not specific to any tissue type the proposed workflow may find broad utility for both clinical and research purposes in other types of cancer.

## Supplementary Material

Supplementary Figure 1 Visual comparison of output of G1 network trained with MSE loss function vs combined Adversarial and VGG loss functions.

Supplementary Figure 2 Method of choosing discrete frequency bands from hyperspectral SRS measurements.

Supplementary Figure 3 Representative examples of virtual staining

Supplementary Figure 4 Representative examples of failure cases

Supplementary Figure 5 Examples demonstrating the virtual sectioning capabilities of the implemented workflow.

Supplementary Figure 6 Heterogeneity of lipid droplet density distribution for one patient between adjacent glands of the same grade.

Supplementary Table 1 One-way ANOVA table for comparison between the 4 groups showing statistically significant difference of lipid droplet density for at least one group from others.

Supplementary Table 2 Unpaired t-tests between the different grades and histologic classes of importance showing statistically significant differences of lipid droplet density distributions.
