# Supplementary material for: Accelerating Cancer Histopathology Workflows with Chemical Imaging and Machine Learning
Source: Cancer Res Commun. 2023 Sep 18;3(9):1875–87. doi: 10.1158/2767-9764.CRC-23-0226 (PMC10506535; doi:10.1158/2767-9764.CRC-23-0226)
Supplement: Supplementary Figure 1 — Visual comparison of output of G1 network trained with MSE loss function vs combined Adversarial and VGG loss functions. [file crc-23-0226-s01.pdf]

Supplementary Figure 1

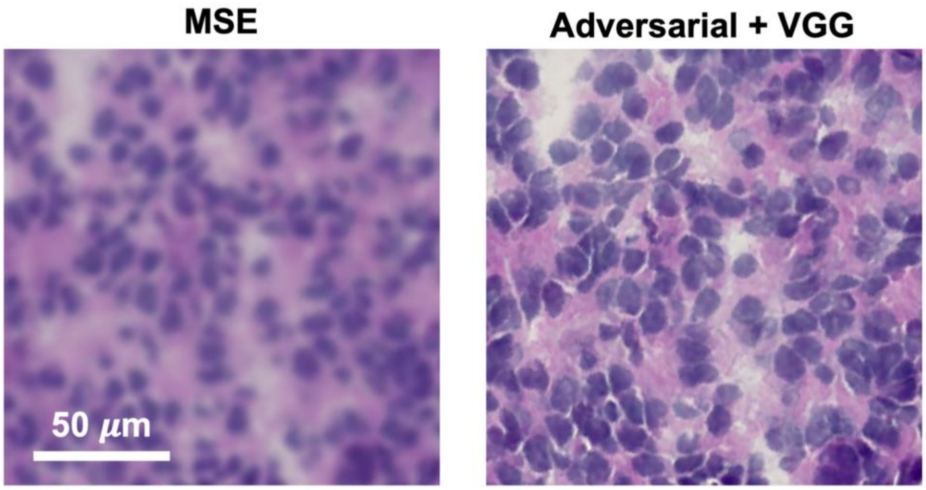

Supplementary Figure 1. **Visual comparison of G1's output using MSE loss function vs Adversarial and VGG loss.** MSE loss results are typically overly smooth and lack important morphological details.
