# Supplementary material for: Accelerating Cancer Histopathology Workflows with Chemical Imaging and Machine Learning
Source: Cancer Res Commun. 2023 Sep 18;3(9):1875–87. doi: 10.1158/2767-9764.CRC-23-0226 (PMC10506535; doi:10.1158/2767-9764.CRC-23-0226)
Supplement: Supplementary Figure 2 — Method of choosing discrete frequency bands from hyperspectral SRS measurements. [file crc-23-0226-s02.pdf]

Supplementary Figure 2

Since the PCa samples were large biopsies (~2cm x 2cm) imaged at 0.5 um or 1um pixel size with approximately 50-100 microsecond pixel dwell time ,imaging the entire highwavenumber frequency region (spanning 2800 cm^{-1} to 3200 cm^{-1} at ~ 4cm^{-1} resolution) can be time-consuming. Thus, we needed to identify the bands that are required with minimum loss in information. To do so, we tested the how much information loss will we have for reconstruction of the full spectra from that at few chosen frequencies. It is important to note that we are not claiming accurate spectral reconstruction, instead, we wanted to see how accurate the spectral reconstruction will be if we added a new band. We are showing the results of the reconstruction in Fig. SI1. In Fig. SI1A and B we demonstrate the reconstruction of 2868 cm^{-1} based on one and five bands respectively. To visually compare the quality of predictions, we show real 2868 cm^{-1}in Fig. SI1C. In Fig. 2SID and E, we show the reconstructed spectra based on one and five bands respectively. The order of selected bands for reconstruction model has been proveided in Fig. SI1F. In Fig. SI1G, we show the RMSE value versus the number of bands input to the reconstruction model. As expected, by adding more bands, the reconstruction gets more accurate. The reconstruction model is an artificial neural network (ANN) with 10 nodes in hidden layer and hyperbolic tangent activation function for the last layer.

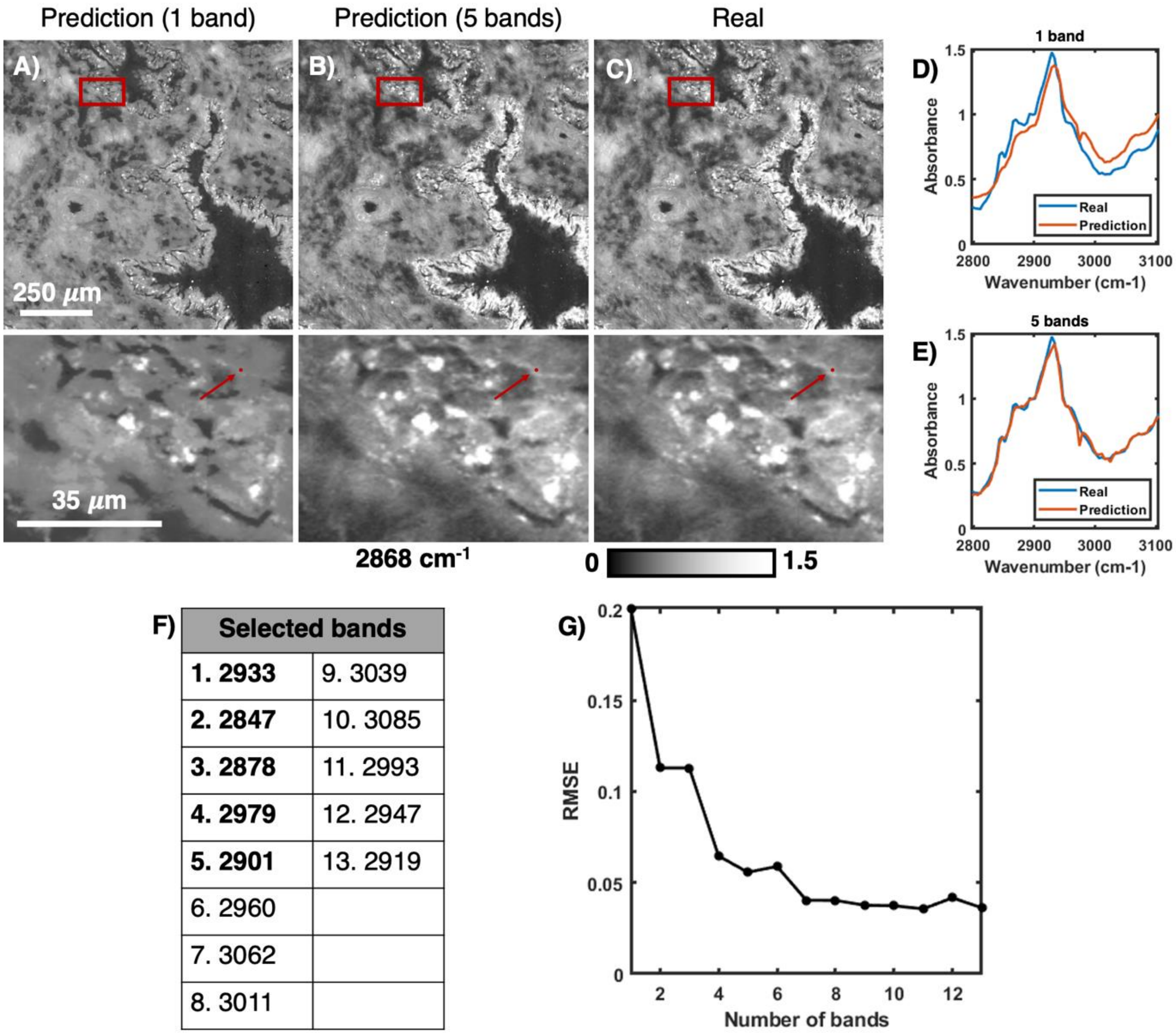

Selected bands	
**1. 2933**	9. 3039
**2. 2847**	10. 3085
**3. 2878**	11. 2993
**4. 2979**	12. 2947
**5. 2901**	13. 2919
6. 2960	
7. 3062	
8. 3011	

Supplementary Figure 2. Wavelength Selection. Hyperspectral data can be reconstructed with 5-7 bands which are 2933, 2847, 2878, 2979, 2901, 2960 and 3062 cm^{-1}. A, Reconstructed 2868 cm^{-1} based

on 1 band (2933 cm^{-1}). **B,** Reconstructed 2868 cm^{-1} based on 5 bands (highlighted in the table). **C**, Real 2868 cm^{-1}. **D,** Reconstructed spectra based on 1 ban. **E,** Reconstructed spectra based on 5 bands. **F,** Order of selected bands for training the reconstruction model. **G,** RMSE value vs number of bands, more RMSE value indicates more deviation from ground truth.
