# Supplementary material for: Accelerating Cancer Histopathology Workflows with Chemical Imaging and Machine Learning
Source: Cancer Res Commun. 2023 Sep 18;3(9):1875–87. doi: 10.1158/2767-9764.CRC-23-0226 (PMC10506535; doi:10.1158/2767-9764.CRC-23-0226)
Supplement: Supplementary Figure 3 — Representative examples of virtual staining [file crc-23-0226-s03.pdf]

Supplementary Figure 3

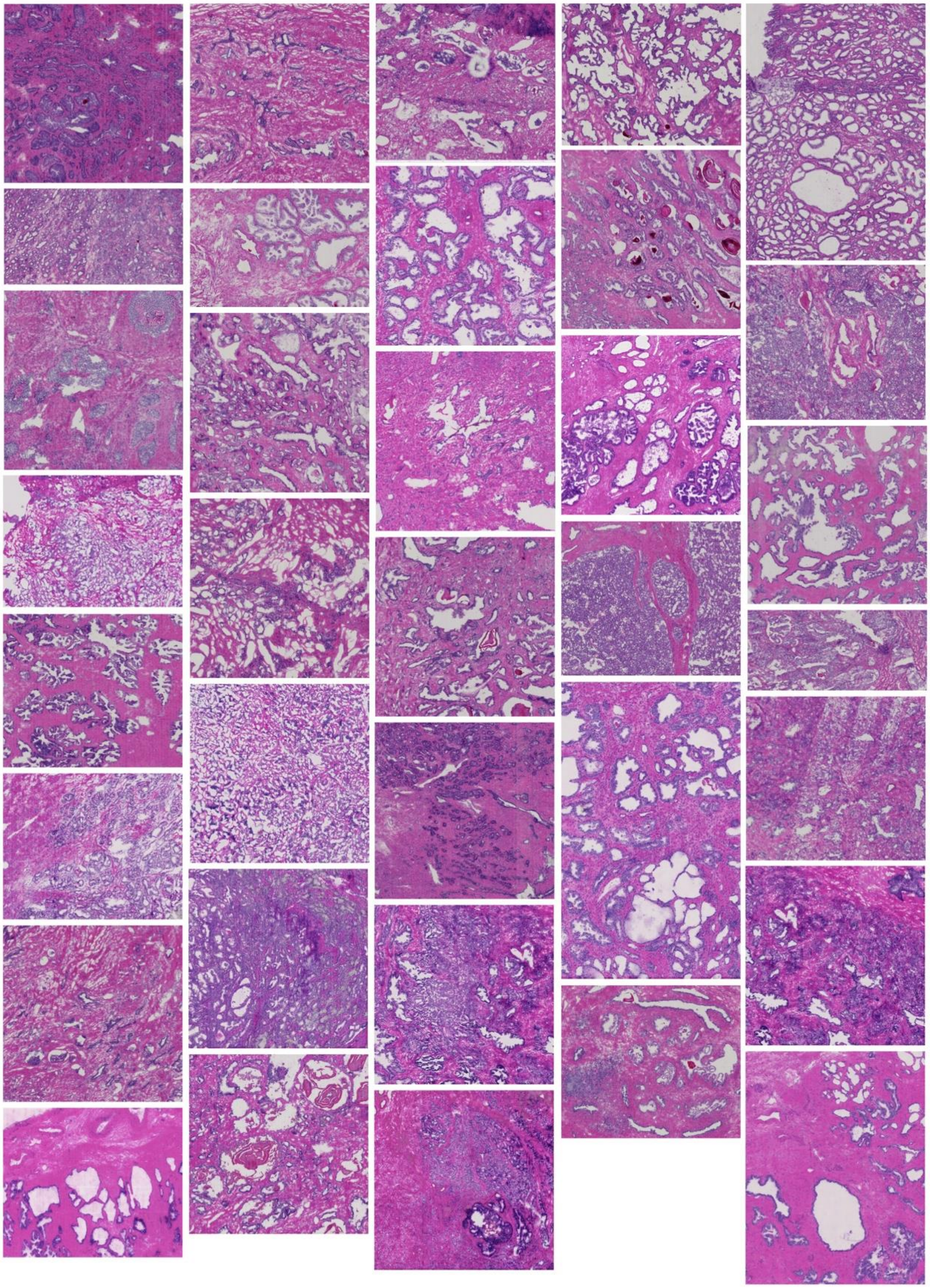

Supplementary Figure 3. More examples of virtual staining for ROIs that have been imaged. We demonstrate 35 ROIs in this figure.
