# Supplementary material for: Accelerating Cancer Histopathology Workflows with Chemical Imaging and Machine Learning
Source: Cancer Res Commun. 2023 Sep 18;3(9):1875–87. doi: 10.1158/2767-9764.CRC-23-0226 (PMC10506535; doi:10.1158/2767-9764.CRC-23-0226)
Supplement: Supplementary Figure 4 — Representative examples of failure cases [file crc-23-0226-s04.pdf]

Supplementary Figure 4

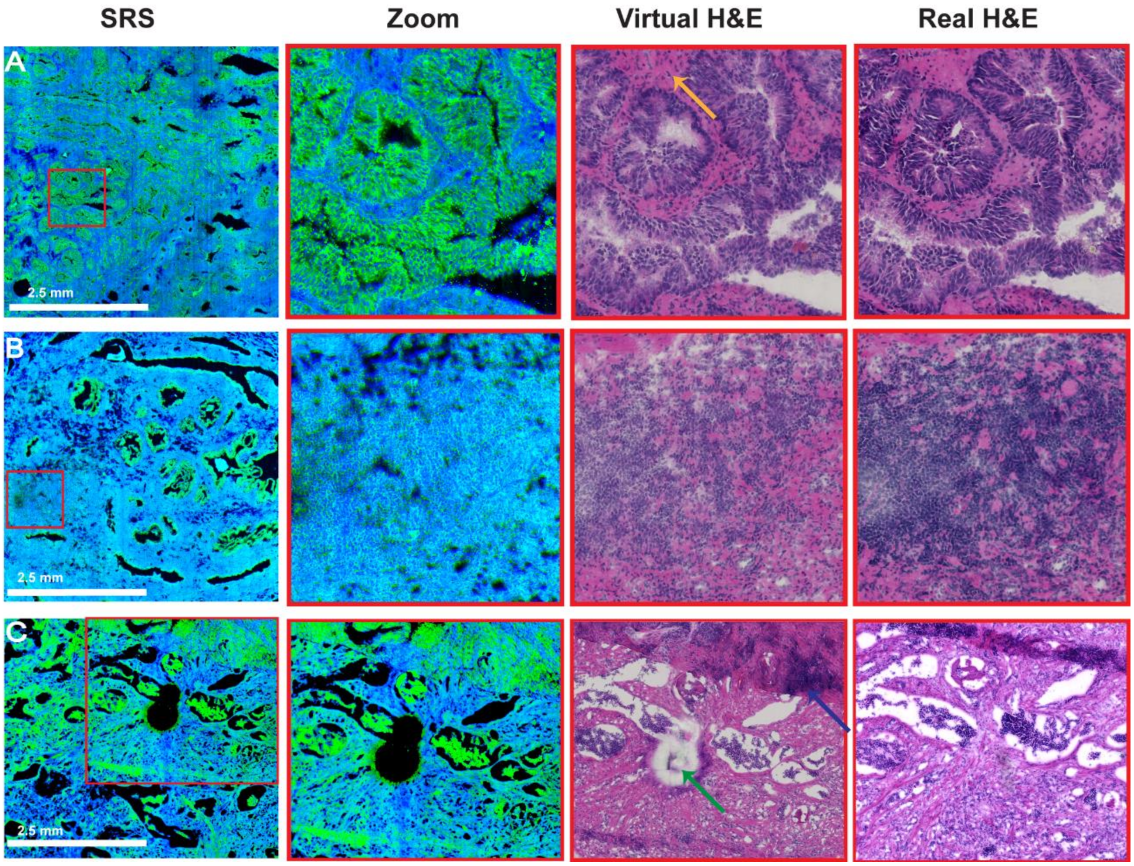

Supplementary Figure 4. **Representative examples of failure cases. A,** Virtual H&E showing more nuclei than real H&E shown with yellow arrow. **B,** The level of inflammation is not always accurately captured. **C,** Artifacts in virtual stain due to photodamage in tissue (green arrow) and folding (blue arrow).
