# Supplementary material for: Accelerating Cancer Histopathology Workflows with Chemical Imaging and Machine Learning
Source: Cancer Res Commun. 2023 Sep 18;3(9):1875–87. doi: 10.1158/2767-9764.CRC-23-0226 (PMC10506535; doi:10.1158/2767-9764.CRC-23-0226)
Supplement: Supplementary Figure 5 — Examples demonstrating the virtual sectioning capabilities of the implemented workflow. [file crc-23-0226-s05.pdf]

Supplementary Figure 5

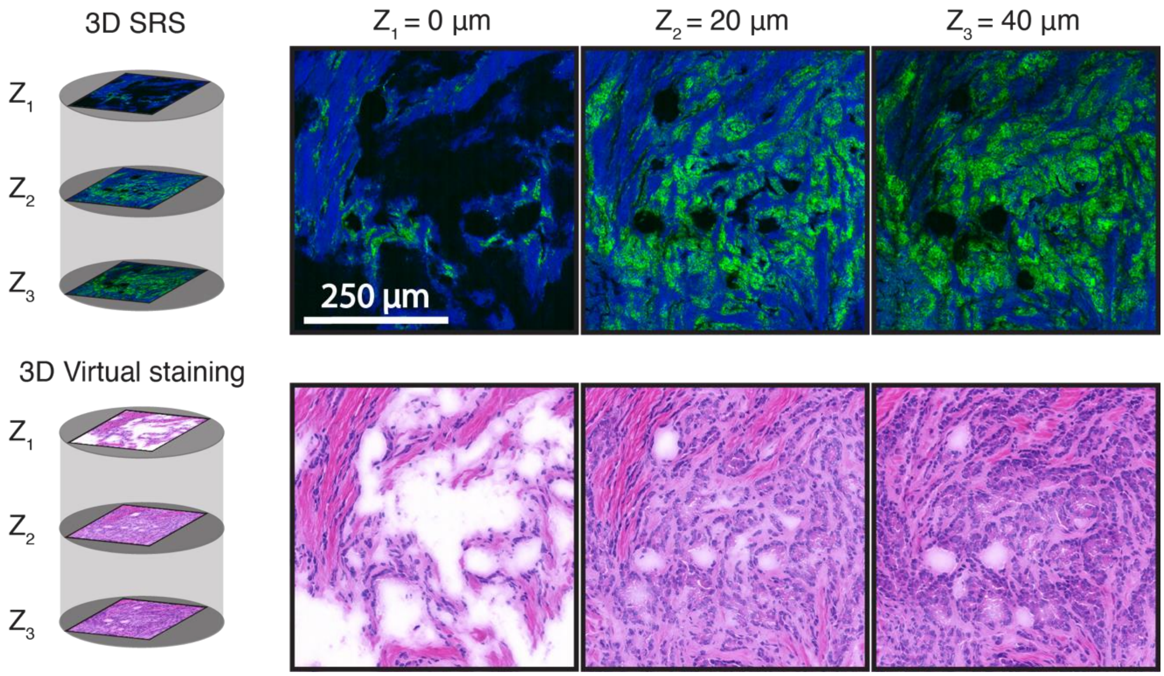

Supplementary Figure 5. Virtual sectioning. Virtual staining of thick prostate samples using 3D SRS imaging, which obviates the need for physical sectioning of samples.
