# Supplementary material for: Accelerating Cancer Histopathology Workflows with Chemical Imaging and Machine Learning
Source: Cancer Res Commun. 2023 Sep 18;3(9):1875–87. doi: 10.1158/2767-9764.CRC-23-0226 (PMC10506535; doi:10.1158/2767-9764.CRC-23-0226)
Supplement: Supplementary Figure 6 — Heterogeneity of lipid droplet density distribution for one patient between adjacent glands of the same grade. [file crc-23-0226-s06.pdf]

Supplementary Figure 6

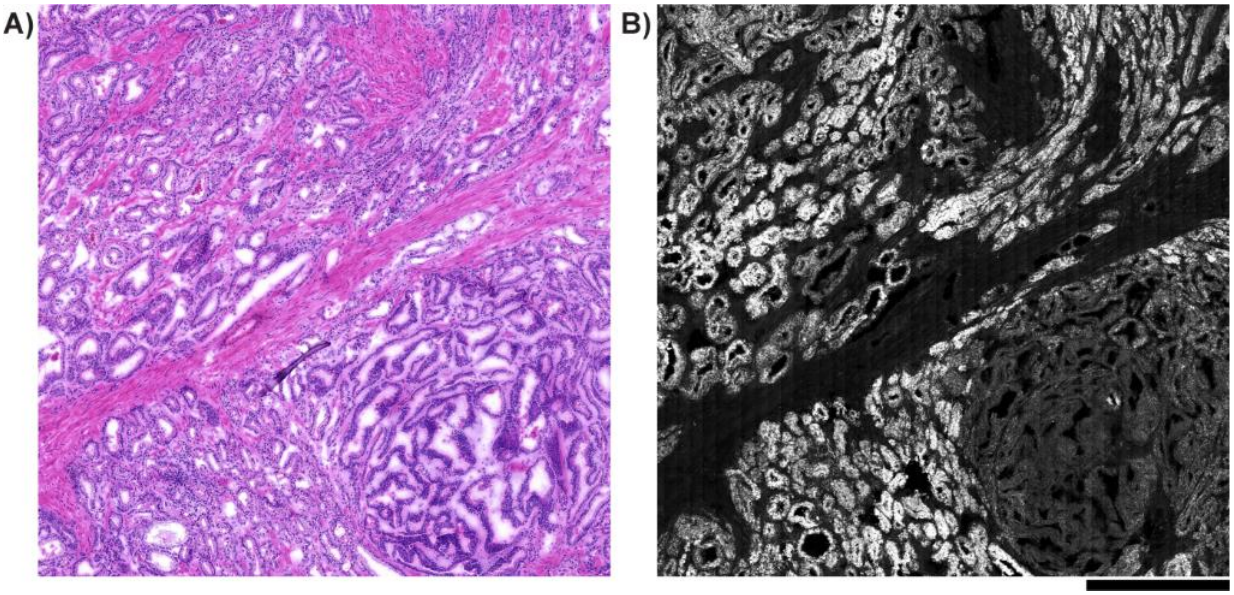

Supplementary Figure 6. Heterogeneity of lipid droplet density for a patient: A) H&E image and B) SRS image corresponding to the lipid channel (ω=2848 cm^{-1}) of tissue sections adjacent to (A) Scale bar in all the images is 500 μm. The red and green arrows point towards area of high and low lipid droplet density in the same patient of tumor of similar grade.
