# Supplementary material for: Accelerating Cancer Histopathology Workflows with Chemical Imaging and Machine Learning
Source: Cancer Res Commun. 2023 Sep 18;3(9):1875–87. doi: 10.1158/2767-9764.CRC-23-0226 (PMC10506535; doi:10.1158/2767-9764.CRC-23-0226)
Supplement: Supplementary Table 1 — One-way ANOVA table for comparison between the 4 groups showing statistically significant difference of lipid droplet density for at least one group from others. [file crc-23-0226-s07.pdf]

Supplementary Table 1

Supplementary Table 1. One-way ANOVA table for comparison of lipid droplet density between the 4 classes showing that at least one group is significantly different from others.

Source	SS	df	MS	F	**ρ>F**
Groups	932.48	3	310.828	129.62	2.19011e-55
Error	786.53	328	2.398		
Total	1719.01	331			
