# Supplementary material for: Accelerating Cancer Histopathology Workflows with Chemical Imaging and Machine Learning
Source: Cancer Res Commun. 2023 Sep 18;3(9):1875–87. doi: 10.1158/2767-9764.CRC-23-0226 (PMC10506535; doi:10.1158/2767-9764.CRC-23-0226)
Supplement: Supplementary Table 2 — Unpaired t-tests between the different grades and histologic classes of importance showing statistically significant differences of lipid droplet density distributions. [file crc-23-0226-s08.pdf]

Supplementary Table 2

Supplementary Table 2. Unpaired t-tests reveal that lipid droplet density of all classes are significantly different with the lowest significant value between high and low.

Stroma-Benign	Stroma-Low	Stroma-High	Benign-High	Benign-Low	High-Low
O(ρ)=E-35	O(ρ)=E-42	O(ρ)=E-30	P=0.0188	O(ρ)=E-7	ρ=0.0298
